# Therapeutic utility of mesenchymal stromal cell (MSC)-based approaches in chronic neurodegeneration: a glimpse into underlying mechanisms, current status, and prospects

**DOI:** 10.1186/s11658-022-00359-z

**Published:** 2022-07-16

**Authors:** Mohaddeseh Rahbaran, Angelina Olegovna Zekiy, Mahta Bahramali, Mohammadsaleh Jahangir, Mahsa Mardasi, Delaram Sakhaei, Lakshmi Thangavelu, Navid Shomali, Majid Zamani, Ali Mohammadi, Negin Rahnama

**Affiliations:** 1 Biotechnology Department, National Institute of Genetic Engineering and Biotechnology (NIGEB), Tehran, Iran; 2 Department of Prosthetic Dentistry, I. M. Sechenov First Moscow State Medical University (Sechenov University), Moscow, Russia; 3 Biotechnology Department, University of Tehran, Tehran, Iran; 4 Faculty of Medicine, Iran University of Medical Sciences, Tehran, Iran; 5 Biotechnology Department, Faculty of Life Sciences and Biotechnology, Shahid Beheshti University, Tehran, Iran; 6 School of Medicine, Sari Branch, Islamic Azad University, Sari, Iran; 7 Department of Pharmacology, Saveetha Dental College, Saveetha Institute of Medical and Technical Science, Saveetha University, Chennai, India; 8 Immunology Research Center, Tabriz University of Medical Sciences, Tabriz, Iran; 9 Department of Medical Laboratory Sciences, Faculty of Allied Medicine, Infectious Diseases Research Center, Gonabad University of Medical Sciences, Gonabad, Iran; 10 Department of Neurology, Imam Khomeini Hospital, Urmia University of Medical Sciences, Urmia, Iran; 11 Department of Internal Medicine and Health Services, Semnan University of Medical Sciences, Semnan, Iran

**Keywords:** Mesenchymal stromal cells (MSCs), Neurotrophic factors (NTFs), Neuroprotection, Differentiation, Neuroinflammation

## Abstract

Recently, mesenchymal stromal cell (MSC)-based therapy has become an appreciated therapeutic approach in the context of neurodegenerative disease therapy. Accordingly, a myriad of studies in animal models and also some clinical trials have evinced the safety, feasibility, and efficacy of MSC transplantation in neurodegenerative conditions, most importantly in Alzheimer’s disease (AD), Parkinson’s disease (PD), amyotrophic lateral sclerosis (ALS), and Huntington’s disease (HD). The MSC-mediated desired effect is mainly a result of secretion of immunomodulatory factors in association with release of various neurotrophic factors (NTFs), such as glial cell line-derived neurotrophic factor (GDNF) and brain-derived neurotrophic factor (BDNF). Thanks to the secretion of protein-degrading molecules, MSC therapy mainly brings about the degradation of pathogenic protein aggregates, which is a typical appearance of chronic neurodegenerative disease. Such molecules, in turn, diminish neuroinflammation and simultaneously enable neuroprotection, thereby alleviating disease pathological symptoms and leading to cognitive and functional recovery. Also, MSC differentiation into neural-like cells in vivo has partially been evidenced. Herein, we focus on the therapeutic merits of MSCs and also their derivative exosome as an innovative cell-free approach in AD, HD, PD, and ALS conditions. Also, we give a brief glimpse into novel approaches to potentiate MSC-induced therapeutic merits in such disorders, most importantly, administration of preconditioned MSCs.

## Introduction

Neurodegenerative disorders are largely characterized by the progressive loss of neural populations that are particularly vulnerable, in contrast with the select static neuronal loss due to toxins or metabolic diseases. Proteopathies are the most prominent neurodegenerative disease, including Alzheimer’s disease (AD), Parkinson’s disease (PD), amyotrophic lateral sclerosis (ALS), and Huntington’s disease (HD) [1, 2]. Additionally, neurodegenerative diseases could be categorized on the basis of ultimate clinical characteristics (for instance, dementia, Parkinsonism, or motor neuron disease), anatomical distribution of neurodegeneration [for example, frontotemporal degeneration (FTD), extrapyramidal disorders, and spinocerebellar degeneration], and primary molecular abnormalities [3, 4]. The existing gold standard for diagnosis is an autopsy-based neuropathological analysis. Of course, particular protein aggregates as well as anatomical vulnerability are often used to characterize neurodegenerative diseases. According to literature, numerous essential procedures correlated with neurodegenerative diseases share progressive neuronal dysfunction and death, including proteotoxic stress and associated abnormalities in the ubiquitin–proteasomal and autophagosomal/lysosomal systems, oxidative stress, programmed cell death (PCD), and neuroinflammation [5–7]. Such disorders show distinct neural pathologies entirely, and the particular pathways for neuronal death are multidimensional, making it impossible to determine and design an efficient and practical treatment strategy [8]. The number of people affected by neurodegenerative diseases and socially critical medical issues is expected to increase sharply in the coming years as the population ages, highlighting the importance of evolvement of novel and more effective therapeutic approaches.

**Fig. 1:**
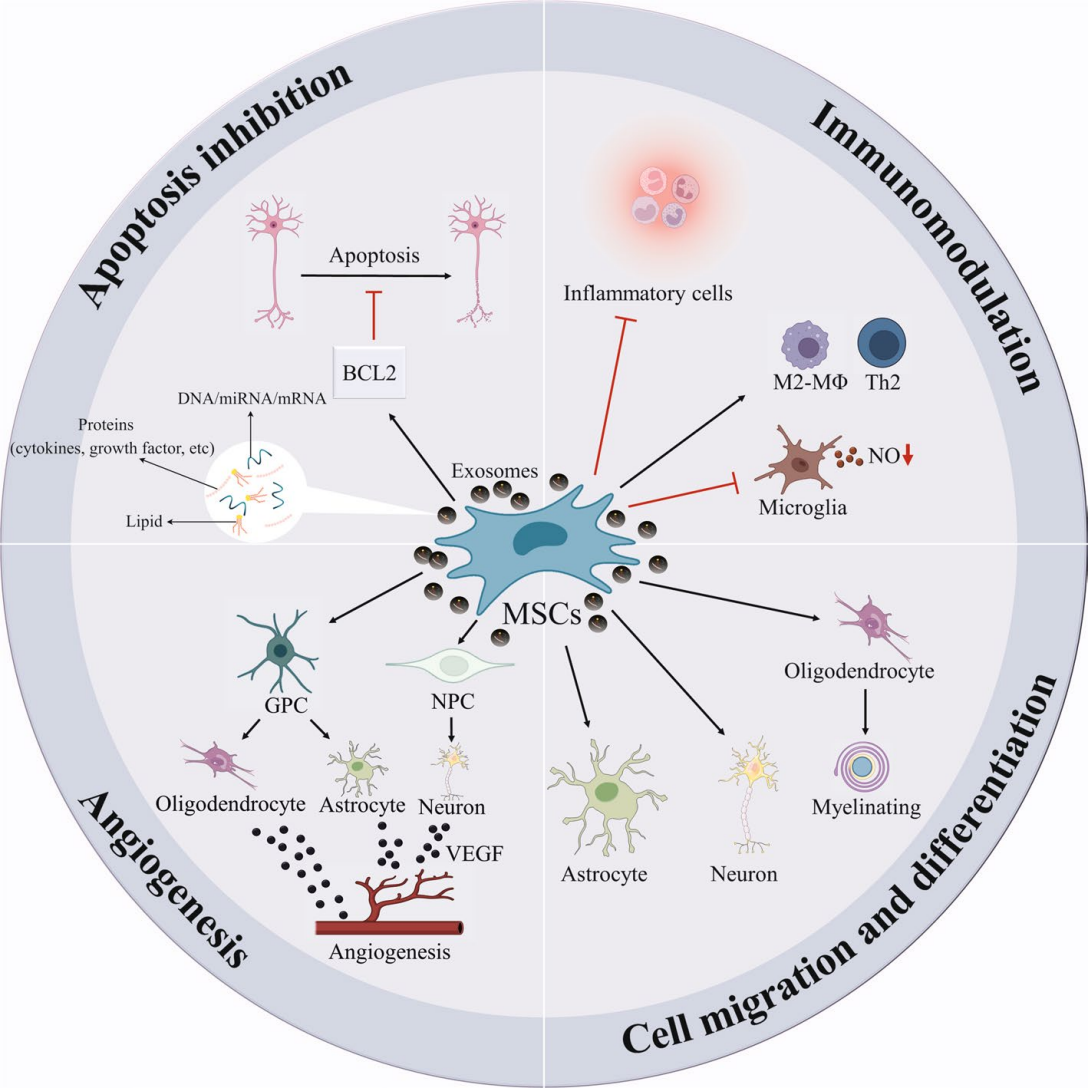
Underlying mechanisms contributing to mesenchymal stromal cell (MSC)-mediated neuroprotection and neurogenesis in chronic neurodegenerative conditions. GPCs, glial progenitor cells; NPCs, neural progenitor cells; VEGF, vascular endothelial growth factor; M2-Mɸ, M2 macrophages; Th2, T-helper 2; NO, nitric oxide; BCL2, B-cell lymphoma 2

Neurodegenerative disease treatment has been a turning point in the history of stem cell therapy throughout the previous four decades, in the 1980s in Mexico, when stem cell treatments for patients with Parkinson’s disease (PD) showed some encouraging outcomes [9]. Nowadays, stem cell technology is noted as an exciting and feasible technique for treating PD, HD, AD, and ALS [10]. Meanwhile, mesenchymal stromal cells (MSCs) have enormous potential for cell therapy since they can be effectively isolated from adult tissue, ex vivo cultivated in culture, and transplanted in an autologous or allogeneic and safe way [11–14]. MSCs have also been observed to be capable of differentiating toward neural fates and secreting a variety of molecules (e.g., growth factors) that, in turn, aid nervous tissue preservation and repair (Fig. 1) [15, 16]. There are several beneficial impacts of human MSC transplantation into rodent models of neurodegenerative disorders that have been identified, surrounding neurotropic factor-mediated neuroprotection, increased neurogenesis, dampened inflammation, and elimination of aberrant protein aggregates [17–19]. Also, MSC-derived exosomes have received increasing interest recently as a novel cell-free strategy for overcoming the problems associated with the direct use of MSCs in the context of regenerative medicine. MSC-derived exosomes contain a wide range of cytoplasmic and membrane proteins, including receptors, enzymes, transcription factors, lipids, extracellular matrix (ECM) proteins, and nucleic acids [20–22]. The contents of these exosomes may influence a wide range of biological processes in cells, including cell reproduction, migration, apoptosis, and immunomodulation, and thereby deliver great competence to restoring neurodegenerative disease-associated deficits [23–25].

This review aims to provide an insight into current research to understand the underlying mechanisms exerted by MSCs for treating neurodegenerative diseases. The other goal is to describe the most current preclinical and clinical research on MSC treatment in neurodegenerative diseases, focusing on in vivo reports.

## Pathophysiology of neurodegenerative diseases

As cited, neurodegenerative diseases develop as a result of advanced deterioration of particular types of neurons responding to each disease [26]. AD, which is described as the main underlying cause of dementia, is largely recognized by advanced cognitive impairment and can also target several regions in the brain that contribute to learning and memory, most importantly the hippocampus (HPC) and medial prefrontal cortex (mPFC) [27]. On the basis of pathological analysis, amyloid and tau (τ) play central roles in AD pathology. Meanwhile, amyloid pathogenesis is characterized by impairment in cleavage of amyloid precursor protein (APP) by β-secretases (BACE1) and γ-secretases, leading to the generation of insoluble Aβ fibrils [28]. Then, the spreading of Aβ oligomers into synaptic clefts fences transmission of synaptic signaling, and finally such amyloid fibrils are polymerized and create plaques. After that, kinase activation facilitates the hyperphosphorylation of microtubule-associated τ protein, causing the establishment of fibrillary and enormously insoluble spots, termed neurofibrillary tangles (NFTs) [29]. The NFTs eventually trigger neuronal cell apoptosis and thereby ease the recruitment of microglia around the lesion site. These events elicit local inflammatory reactions and neurotoxicity [30]. Further, PD is identified by gradual deterioration of nigrostriatal dopaminergic innervation, causing tremor, rigidity, postural instability, and bradykinesia [31]. The most critical diagnosis comprises the detection of α-synuclein-enclosing fibrillar aggregates called Lewy bodies (LBs) or Lewy neurites by histopathological analysis. Owing to the destruction of cells adjacent to LBs, these constructions are considered a marker for neuronal deterioration [32]. However, alteration in the expression profile of several genes may stimulate dopaminergic neuron loss [33]. Correspondingly, it appears that genetic mutations in a group of genes, such as Parkins, in association with environmental insults, cause dopaminergic neuron loss. Mutations in such genes are accountable for approximately 50% of PD familial cases and demonstrate either autosomal dominant or autosomal recessive inheritance [34]. Also, HD, a well-known incurable hereditary neurodegenerative condition, causes motor impairment and cognitive decline because of the mutated and toxic huntingtin (HTT) protein function [35]. Thanks to the central role of HTT protein in adjusting neuronal progress, disruption in HTT expression and normal function may lead to HD progress. Meanwhile, augmentation in CAG repeats numbers within the huntingtin (HTT) gene gives rise to the extension of the polyglutamine tract in the amino terminus of the HTT protein and, in turn, engenders toxic HTT protein [36]. The toxic HTT proteins can strikingly target striatal medium spiny neurons, supporting their loss and subsequent neuroinflammation. Other studies have evinced that a single-nucleotide polymorphism (SNP) in nuclear factor-κB (NF-κB) binding site in the promoter of the HTT gene may provoke HD onset [37]. Besides, ALS is characterized by gradual loss of motor neurons (MNs) in the brainstem and muscle denervation atrophy in association with gliosis, induction of microglial activation along with the cytoplasmic assemblages of TAR DNA-binding protein 43 (TDP-43), and superoxide dismutase (SOD1) [38]. As a result of the detection of cytoplasmic inclusions containing TDP-43 and fused in sarcoma (FUS) pathology in patients with ALS, ALS pathogenesis depends primarily on RNA processing. Such genes participate in cytoskeletal dynamics, pre-mRNA splicing, RNA transport, and RNA translation [39]. Also, the potent role of extension of noncoding GGGGCC hexanucleotide repeat in the chromosome 9 open reading frame 72 (C9ORF72) gene has been proven in patients with ALS [40].

## The rationality of MSC therapy in neurodegenerative diseases

### MSC differentiation into neural cell lineages

Few studies indicate that transplantation of MSC-derived neural cells could stimulate favored effects in vivo [41–43]. In this regard, intracerebral administration of umbilical cord (UC)-MSC-derived neural cells reduced Aβ deposition and concomitantly restored memory impairment in AD mice [42]. The desired effects are likely caused by induction of M2-like microglia activation and alleviation of neuroinflammation [42]. In 2019, Wei et al. also found that administration of umbilical-cord (UC)-MSCs-derived cholinergic-like neurons genetically modified to overexpress brain-derived neurotrophic factor (BDNF) resulted in ameliorated spatial learning and memory competencies in rats with AD [44]. Also, transplantation led to the promoted secretion of acetylcholine in the hippocampus, boosted both astrocyte and microglia activation, attenuated levels of Aβ, reserved neuronal loss, and finally potentiated neurogenesis [44]. On the other hand, MSC in vivo differentiation into the neural cell post-transplantation has partially been evidenced [45, 46]. In the 1-methyl-4-phenyl-1,2,3,6-tetrahydropyridine (MPTP)-induced monkey model of PD, intrastriatal injected endometrium-derived mesenchymal stromal cells (EDSCs) demonstrated neuron-like morphology, expressed tyrosine hydroxylase (TH), and eventually augmented the frequency of TH-expressing cells in vivo. Likewise, in a neurotoxin 6-hydroxydopamine (6-OHDA)-induced rat model of PD, injected BM-MSCs by intranigral route partially differentiated into nestin- and GFAP-expressing cells, improving behavior abnormalities in transplanted PD rats [46]. Notwithstanding, MSC’s capability to generate fully functional neurons in vivo has not yet been proven [47]. It has been suggested that MSC transdifferentiation into neuronal cells has no remarkable effect on neural tissue recovery in neurological disease [48]. Thus, MSC-secreted molecules are thought to be responsible for exerting the favored effects in vivo.

### Immunomodulatory competencies

Neuroinflammation involves various chronic, pro-inflammatory, immune system-mediated processes, mostly allied with neurodegeneration [49]. These immunological processes underlie the development of different neurodegenerative diseases. Upregulated microglia and astrocyte activation in association with higher levels of pro-inflammatory mediators has been found in patients with PD, ALS, HD, and AD as a result of neural cell apoptosis [50]. In addition to the damage to neural tissue, neuroinflammation hinders activation of endogenous brain repair mechanisms, underlining the importance of inhibiting the neuroinflammatory process [47]. Meanwhile, microglia-elicited inflammation plays a decisive role in the pathogenesis of several neurodegenerative diseases [51]. Owing to their remarkable immunomodulatory attributes, MSC therapy can be a rational plan to compromise inflammatory and immune response by secretion of a myriad of soluble mediators, thus protecting the neural cell. MSC downregulated immune responses in a traumatic brain injury (TBI) model by induction of tumor necrosis factor α (TNFα)-stimulated gene/protein 6 (TSG-6) expression, and subsequently hindered microglia activation [52, 53]. Also, Min et al. showed that MSC secretome elicited a potent anti-inflammatory effect in a subarachnoid hemorrhage (SAH) rat model by the polarization of microglia to the anti-inflammatory M2 phenotype as well as reduction of pro-inflammatory cytokines levels in both parietal cortex and hippocampus [54]. Other studies also revealed that MSC transplantation could suppress NLR family pyrin domain containing 3 (NLRP3) expression and inhibit inflammation by stimulating M2 microglial activation in vivo [55, 56]. Similarly, systemic administration of MSC-derived exosome downregulated expression of pro-inflammatory cytokine TNF-α, interleukin (IL)-1β, and IL-6, but upregulated anti-inflammatory IL-10, IL-4, and IL-13 in cortex and hippocampus of AD mice [57]. These alterations in expression patterns of pro-and anti-inflammatory cytokines could ultimately restore learning and memory deficits and attenuate Aβ levels [57]. In the SOD1^G93A^ transgenic mouse model of ALS, other studies also showed that intramuscular [58] and intraventricular [59] administration of MSCs could hinder disease progress by downregulating inflammatory inducible nitric oxide synthase (iNOS) activation [58] and suppressing expression of pro-inflammatory cytokines in vivo [59].

Although MSCs of various origins alleviate neuroinflammation, the administration of autologous blood- or adipose tissue (AT)-derived MSCs appears mostly preferable as these cells can be procured from the patient at any time. Conversely, placenta- or umbilical cord (UC)-derived MSCs should be isolated and stored for potential future use. Importantly, the low immunogenicity of MSCs facilitates using allogeneic cells from general cell banks [60].

### Secretion of neurotrophic factors

**Fig. 2:**
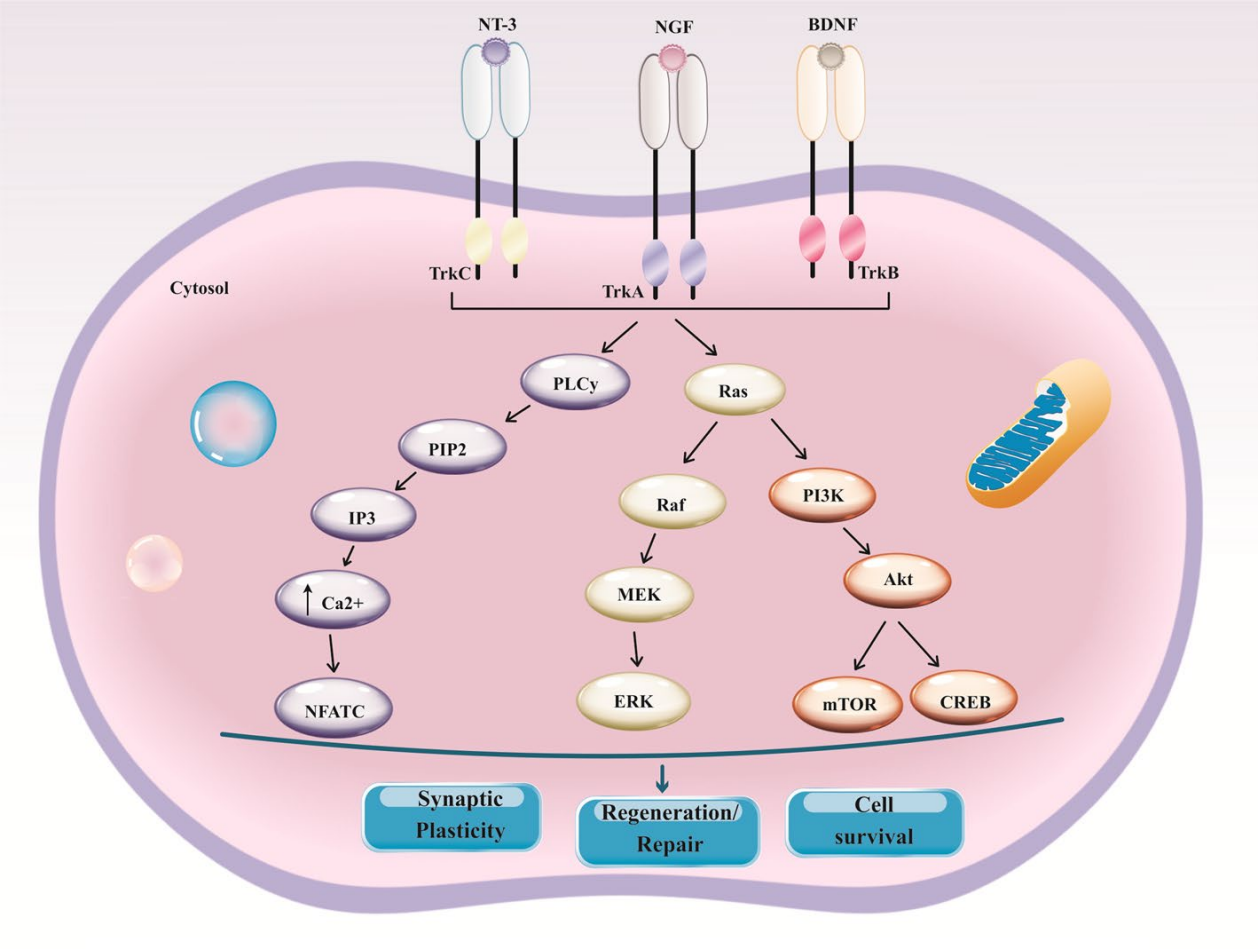
NTF signaling through Trk receptors. This diagram shows the main intracellular signaling axes associated with each neurotrophin receptor. Each Trk receptor isoform connects to a mature NTF and performs through three predominant pathways, including Ras/MEK/ERK, PLC-γ1/PKC, and PI3K/Akt. TrkA, TrkB, and TrkC, tropomyosin receptor kinases; NTFs, neurotrophic factors; mek, mitogen-activated protein kinase; ERK, extracellular-signal-regulated kinase; PLCγ1, phospholipase C gamma 1; PI3K, phosphatidylinositol 3-kinase; PKC, protein kinase C; NFATC, nuclear factor of activated T cells, cytoplasmic 1; CREB, cAMP response element-binding protein; mTOR, mechanistic target of rapamycin; PIP2, phosphatidylinositol 4,5-bisphosphate

A large number of studies have implied that functional improvement in animal models of neurodegenerative diseases following MSC administration may potently arise from improved levels of neurotrophic factors (NTFs) in the brain, facilitating neuroprotection and neurogenesis, inhibition of oxidative stress, and eventually downregulation of the inflammatory response [61–65]. NTFs, in particular, nerve growth factor (NGF), brain-derived neurotrophic factor (BDNF), and neurotrophin-3 (NT-3), trigger signaling axes such as PI3K/Akt and ERK, leading to improved neural cell survival and plasticity (Fig. 2). In the AD mouse model, UC‐derived MSCs could restore cognitive impairment and also support neural network secretion by hepatocyte growth factor (HGF) and resultant induction of the cMet/AKT/ glycogen synthase kinase-3β (GSK-3β) signaling axis in the hippocampus [61]. Similarly, umbilical cord blood (UCB)-derived MSCs heightened endogenous hippocampal regeneration and inspired synaptic activity by secretion of growth/differentiation factor-15 (GDF-15) following intrathecal injection into APP/PS1 mice [62]. Likewise, in the striatum of MPTP-induced mice, systemic administration of AT-MSC restored dopamine transporter expression and stimulated functional recovery mainly by up-regulation of expression of NTFs, such as BDNF and GDNF [63]. Also, transplanted UCMSC markedly diminished gliosis, sustained motor coordination as well as muscle activity, and finally potentiated striatal volume and dendritic length in the mitochondrial toxin 3-nitropropionic acid (3-NP)-induced rat model of HD [66]. Notably, these beneficial effects were likely related to the ability of MSCs to release paracrine factors, such as GDNF and vascular endothelial growth factor (VEGF) [66].

Apart from the original capability of MSCs to produce NTFs, recent reports have focused on the modification of MSCs to overexpress NTFs to entice more favored therapeutic outcomes in vivo. Accordingly, administration of adipose tissue (AT)-MSC-overexpressing GDNF into 6-OHDA mouse model of PD [67], BM-MSC-overexpressing VEGF into APP/PS1 mouse model of AD [68], BM-MSC-overexpressing BDNF into an Aβ rat model of AD [69], MPTP monkeys model of PD [70], and YAC128 mice model of HD [71], and finally injection of UC-MSC-overexpressing BDNF into SOD1^G93A^ mouse model of ALS [72] resulted in promising outcomes in vivo.

Together, it has been evidenced that paracrine release from MSCs enables potentiated neurogenic capacity and resultant functional improvement. Nonetheless, we cannot exclude the possibility of direct influence of NTF and other biomolecules on multiple cellular processes including homeostasis, reduction in reactive oxygen species (ROS) generation, negative regulation of inflammation, and neuronal survival [47].

### Secretion of other biomolecules

Among the therapeutic capacities of MSCs, the angiogenic ones have been broadly investigated by virtue of their importance in various pathological conditions such as brain injury. Angiogenesis, as a natural defense mechanism, aids in restoring oxygen and nutrient supply to the damaged brain tissue upon ischemia or similar conditions. Angiogenesis may support brain perfusion, and enhance neuronal survival, brain plasticity, and neurologic recovery [73]. MSCs produce remarkable levels of vascular endothelial growth factor (VEGF), transforming growth factor-beta (TGF-β), hepatocyte growth factor (HGF), IL-8, basic fibroblast growth factor (bFGF), monocyte chemoattractant protein 1 (MCP-1), and IL-6, as well as various miRNAs with angiogenic function [18, 74]. With respect to the in vivo MSC niche circumstances that occur in tissue injury, hypoxia priming has been considered as the main priming approach to direct MSCs toward a pro-angiogenic phenotype [75].

In addition to direct targeting of immune cells by cell-to-cell contact, MSCs affect them in a paracrine manner by secreting several anti-inflammatory cytokines and chemokine. For instance, TGF-β in MSC-derived exosome exerts anti-inflammatory influences by negative regulation of the nuclear factor (NF)-κB pathway and restoring the TGF-β pathway in lipopolysaccharide (LPS)-stimulated microglia [76]. MSCs also release IL-10 under specific milieu with the inflammatory environment and existence of interferon-gamma (IFN-γ), interleukin-1β (IL-1), and tumor necrosis factor-alpha (TNF-ɑ), inducing particular Toll-like receptors (TLRs) on MSCs [77]. IL-10 downregulates the release of pro-inflammatory cytokines, including TNFα, IL-1, IL-6, IL-8, and IL-12, by dendritic cells (DCs) and also inhibits the expression of MHC II molecules accompanied by co-stimulatory complex B7 on their surface [78]. Irrespective of cytokines, MSCs could secrete various types of chemokines, including CCL2 (MCP-1), CCL3 (MIP-1α), CCL4 (MIP-1β), CCL5 (RANTES), CCL7 (MCP-3), CCL20 (MIP-3α), CCL26 (eotaxin-3), CXCL1 (GROα), CXCL2 (GROβ), CXCL5 (ENA-78), CXCL8 (IL-8), CXCL10 (IP-10), CXCL11 (i-TAC), CXCL12 (SDF-1), and CX3CL1 (fractalkine) [79–81]. Chemokines mediate the interactions between MSCs and other types of immune cells and thus play critical roles during the process of MSC-mediated immunomodulation. For instance, chemokines CXCL9, CXCL10, and CXCL11 induce the migration of T cells in proximity of MSCs, and these cells are targets of the local inhibitory influences of nitrogen oxide (NO) released by the MSCs [82].

## Biogenesis and compositions of MSC-derived exosomes

In 1983, Harding and Johnstone, for the first time, displayed that transferrin receptor accompanied by small 50 nM vesicles were produced and then secreted from maturing blood reticulocytes into the extracellular space by receptor-mediated endocytosis and recycling [83, 84]. Rose Johnstone named these vesicles “exosomes.” Exosomes are a subtype of extracellular vesicle (EV) with a diameter in the range of 40–150 nm. Such vesicles are typically secreted by several sorts of cells, most importantly, stem/stromal cells, immune cells, or tumor cells [85]. Exosomes contain various biological ingredients such as proteins, lipids, mRNAs, and miRNAs as cargo [86, 87]. Upon secretion in a well-organized process, these cargos are conveyed to the recipient cells, and so modify physiological cells, circumventing comprehensions about the direct application of stromal cells [88]. Thanks to their capacities to instigate endogenous neurogenesis and modulate inflammatory responses, exosomes have currently been suggested as a logical therapeutic alternative for neurodegenerative diseases therapy [89].

The generation process of exosome comprises three crucial steps: (1) formation of endocytic vesicles by invagination of the plasma membrane, (2) formation of multivesicular bodies (MVBs) following endosomal membranes’ inward budding, and (3) incorporation of established MVBs with the plasma membrane and secretion of the vesicular contents called exosomes [90, 91]. Recent reports have strongly evidenced that MSC-derived exosomes could stimulate substantial functional recovery and also ameliorate cognitive decline in preclinical models as a result of NTF and antioxidant molecule delivery to adjacent neural cells [92–95].

## Preclinical studies based on MSC therapy in neurodegenerative diseases

### AD

As cited, native (Table 1) and also genetically modified (Table 2) MSC transplantation has become a potential treatment for common neurodegenerative diseases. In vitro, MSC co-culture with Aβ-induced neural cells may lead to the secretion of remarkable rates of pro-inflammatory cytokines such as IL-10 and TGF-β into the culture medium [96]. Moreover, MSCs could improve expression levels of survival-involved mammalian target of rapamycin (mTOR), 5′ AMP-activated protein kinase (AMPK), glycogen synthase kinase-3β (GSK-3β), Wnt3, and β-catenin in preclinical models of AD [96]. Studies on the SAMP8 mouse model of AD also showed that administration of UC-MSC could restore lesioned neural cells, exert functional recovery, and also ameliorate cognitive decline by secretion of HGF [97]. HGF, in fact, inhibited hyperphosphorylated tau, reduced spine damage, and improved synaptic plasticity through the upregulation of the cMet/AKT/GSK3β signaling pathway in vivo [97]. In another study, systemic injection of UC-MSCs also enhanced cognitive function without any change in Aβ levels in the hippocampus in the Tg2576 mouse model of AD [98]. Additionally, stromal cell therapy could inspire a robust attenuation in the level of malondialdehyde (MDA), and conversely promote nitric oxide (NO) levels and superoxide dismutase (SOD) activities in vivo, eliciting antioxidant effects [98]. It seems that improvement in silent information regulator 1 (Sirt1), BDNF, and synaptophysin (SYN) levels in the hippocampus contribute to triggering MSC-mediated cognitive function in AD mice [98]. Sirt1 is a ubiquitously expressed protein that mostly contributes to the downregulation of reactive oxygen species (ROS) and inflammation [99]. Thereby, it appears that MSCs could improve cognitive function mainly by downregulation of oxidative stress accompanied by inspiring hippocampal neurogenesis by secreting neuroprotective factors [98]. Likewise, amniotic mesenchymal stromal cells (AM-MSCs) administration gave rise to decreased Aβ deposition, improvement in hippocampal neurogenesis in the subgranular zone (SGZ), and restored spatial learning and memory deficits in the AD mouse model [100]. Irrespective of the release of BDNF, enhancement of Aβ-degrading factors levels, improvement in microglia activation, and downregulation of neuroinflammation are other fortunate effects that potently play critical roles in this regard [100]. Like direct administration of MSCs, UC-MSC-derived cholinergic-like neurons could enhance spatial learning and memory capability by augmenting the secretion of acetylcholine and choline acetyltransferase (ChAT), improving astrocyte and microglia activation, averting neuronal cell loss as well as triggering neurogenesis [44].

**Table 1:** Native MSC therapy in preclinical models of common neurodegenerative diseases

Condition	Model	Cell source	Administration route	Result	References
AD	APP/PS1 mice	BM	Intraventricular	Restoring cognitive deficits by upregulation of miR-146a and downregulation of NF-κB	[105]
AD	SAMP8 mice	UC	intraperitoneal	Restoring cognitive deficits mediated by HGF in the AD hippocampus following stimulation of cMet/AKT/GSK3β axis	[61]
AD	3xTg mice	UC	Intravenous	Improving the MSCs engraftment and neural recovery by combination therapy with resveratrol	[101]
AD	Rat	ESC	Intraarterial	Suppressing Aβ-induced cell death in the hippocampus in association with provoking the autophagolysosomal clearance of Aβ	[189]
AD	Rat	BM	Intravenous	Attenuation of memory and cognition impairment by melatonin-pretreated MSCs	[103]
AD	Rat	AT	Intravenous	Improving the learning, memory, and cognition by melatonin-pretreated MSCs	[102]
AD	3xTg mice	BM	Intraventricular	Inspiring a population of proliferating cells in the SVZ	[190]
AD	3xTg mice	BM	Intravenous	Alleviating the Tau phosphorylation and inflammation with no effect on Aβ-42 levels	[191]
AD	Amyloid β mice	UCB	Intracerebral	Restoring the learning, memory, and cognition	[192]
AD	5xFAD mice	BM	Intraventricular	Attenuation of learning impairment	[193]
AD	Amyloid β mice	BM	Intracerebral	Reduction in Aβ aggregates and supporting synaptic transmission	[194]
AD	APP/PS1 mice	UC	Intrathecal	Improving the endogenous adult hippocampal neurogenesis as a result of GDF-15 secretion	[62]
AD	5XFAD mice	WJ	Intrahippocampal	Promoting the proteasome activity and reducing the accumulation of ubiquitin-conjugated proteins mediated by MSC-secreted agouti-related peptide (AgRP)	[195]
PD	MPTP mice	BM	Intracranial	Marked synergistic impacts between electric stimulation and MSCs due to the enhanced levels of dopamine and reduced pro-inflammatory cytokines, restoring behavioral function	[196]
PD	MPTP mice	AT	Intravenous	Inducing alteration in dopamine transporter expression, promoting BDNF and GDNF levels in the striatum	[63]
PD	6-OHDA rat	BM	Intracarotid	No effect on motor impairment	[116]
PD	6-OHDA rat	BM	NA	Synergistic effect between G-CSF and MSCs by downregulation of pro-inflammatory cytokines, stimulating antioxidant enzymes and finally augmenting	[123]
PD	MPTP monkey	Endometrial	Intrastriatal	Enhancing the frequency of TH positive cells and also dopamine metabolite concentrations	[45]
PD	MPTP mice	Endometrial	Intracranial	Improvement of dopamine production	[108]
PD	6-OHDA rat	WJ	Intravenous	Restoring dopaminergic neurons and enhancement of the levels of BDNF and NGF	[64]
PD	Rat	BM	Intravenous	Restoring rotational behavior and enhancing TH-positive cell	[65]
PD	Rotenone rat	HED	Intravenous	PD recovery by modifying the cholinergic synapses, calcium signaling pathways, and axon guidance	[197]
PD	6-OHDA rat	BM	Intrastriatal	Improving the viability of striatal/nigral dopaminergic terminals concomitant with inducing neurogenesis in SVZ	[198]
PD	MG-132 rat	BM	Intravenous	Neuroprotective effects on dopaminergic neurons	[199]
PD	6-OHDA rat	BM	Intranigral	Differentiation into nestin-, neuron-specific enolase-, and GFAP-positive cells	[46]
PD	6-OHDA rat	BM	Intrastriatal	Partial rescue of dopaminergic pathway	[200]
PD	MPTP mice	BM	Intravenous	Neuroprotective effects on dopaminergic neurons, reducing blood–brain barrier damage and downregulation of neuroinflammation	[201]
PD	Rotenone rat	BM	Intranasal	Improved locomotor functions	[202]
ALS	SOD1^G93A^ mice	BM	Intravenous	Enhancing motor neuron frequency, and reducing denervation and myofibril atrophy	[203]
ALS	SOD1^G93A^ mice	BM	Intravenous	Augmenting pathological scores	[136]
ALS	SOD1^G93A^ mice	AT	Intravenous	Marked and durable impacts on motor function by improving bFGF and VEGF levels	[135]
ALS	SOD1^G93A^ mice	NA	Intraventricular Intraspinal	Intraspinal administration of MSCs has superiority over intraventricular injection in terms of restoring functional activity	[137]
ALS	SOD1^G93A^ mice	BM	Intraspinal Intravenous	Prolonged lifespan of treated mice	[133]
ALS	SOD1^G93A^ rat	BM	Intracerebospinal	Prolonged lifespan induced by chimerization of the astroglial population in the lumbar spinal cord following stem cell transplantation	[139]
ALS	SOD1^G93A^ mice	Muscle	Intraventricular	Sustained motor function	[134]
ALS	SOD1^G93A^ mice	BM	Intraspinal	Sustained motor function and dampened neuroinflammation	[204]
ALS	SOD1^G93A^ rat	BM	Intrathecal	Neuroprotective effects on motor neurons and neuromuscular junctions by downregulation of apoptosis, necroptosis, and autophagy pathway	[138]
ALS	SOD1^G93A^ mice	UCB	Intramuscular	Downregulation of inflammation by targeting iNOS/NO signaling pathway	[58]
ALS	SOD1^G93A^ mice	UC	Intraventricular	Downregulation of pro-inflammatory cytokine levels, upregulation of anti-inflammatory cytokine levels, and promotion of IGF-1 levels in the lumbar spinal cord	[59]
ALS	SOD1^G93A^ mice	Amniotic	Intravenous	Prolonged survival, restored motor functions, and suppressed neuroinflammation	[205]
HD	Transgenic mice	BM	Intrastriatal	Improving BDNF levels in the striatum	[127]
HD	Transgenic rat	BM	Intrastriatal	Co-transplanting MSCs with NSCs led to more favorable behavioral sparing	[206]
HD	Transgenic mice	BM	Intranasal	Improved therapeutic benefits by MSCs preconditioning with mood stabilizers lithium and valproic acid	[131]
HD	Transgenic mice	UC	Intravenous	Reducing astrogliosis, and neuroinflammation by downregulation of NF-κB p65 phosphorylation	[207]
HD	3-NP rat	UC	intrastriatal	Improving motor function, enhancing striatal volume, and dendritic length in striatum mediated through the production of VEGF and GDNF by MSCs	[66]
HD	QA rat	BM	Intrastriatal	Reduced striatum atrophy	[208]
HD	3-NP rat	BM	Intrastriatal	Improvement in BDNF, collagen type I, and fibronectin levels in brain	[128]
HD	Transgenic mice	UC	Intrastriatal	Partial improvement of spatial memory	[209]
HD	QA rat	BM	Intravenous	Amelioration of motor and cognitive impairment	[210]

MSC, Mesenchymal stromal cells; AD, Alzheimer’s disease; PD, Parkinson’s disease; ALS, amyotrophic lateral sclerosis; HD, Huntington’s disease; BM, bone marrow; UC, umbilical cord; UCB, umbilical cord blood; AT, adipose tissue; ESC, embryonic stem cell; WJ, Wharton’s jelly; 6-OHDA, 6-hydroxydopamine; MPTP, 1-methyl-4-phenyl-1,2,3,6-tetrahydropyridine; 3-NP, 3-nitropropionic acid; QA, quinolinic acid; NF-kB, nuclear factor kappa B; HGF, hepatocyte growth factor; GSK-3β, glycogen synthase kinase-3β; Aβ, amyloid beta peptide; SVZ, subventricular zone; GDF-15, growth/differentiation factor-15; GDNF, glial cell line-derived neurotrophic factor; BDNF, brain-derived neurotrophic factor; TH, thymidine hydroxylase; G-CSF, granulocyte colony-stimulating factor; NGF, nerve growth factor; VEGF, vascular endothelial growth factor; bFGF, basic fibroblast growth factor; iNOS, inducible nitric oxide synthase; IGF-1, insulin like growth factor-1; GFAP, glial fibrillary acidic protein; NSCs, neural stem cells; miRs, microRNAs

**Table 2:** Genetically modified MSC therapy in preclinical models of common neurodegenerative diseases

Condition	Model	Cell source	Gene	Administration route	Results	References
AD	APP/PS1 mice	BM	CX3CL1 Wnt3a	Intraventricular	Amelioration of learning and memory function	[107]
AD	APP/PS1 mice	BM	lin28B	Intraventricular	Stimulation of MSCs proliferation in vivo, reducing cognitive deficits, enhancing the elimination of Aβ, attenuation of microglia activation and neuronal cell death	[106]
AD	APP/PS1 mice	BM	MiR-937	Intrahippocampal	Augmenting brain-4 secretion by MSCs	[211]
PD	6-OHDA mice	BM	Nurr1	Intrastriatal	Improving the frequency of TH-positive cells in SN, suppression of glial cells activation, and downregulation of the expression of pro-inflammatory factors	[119]
PD	6-OHDA mice	AT	GDNF	Intrastriatal	Improving behavioral phenotype	[67]
AD	APP/PS1 mice	BM	VEGF	Intraventricular	Attenuation of cognitive impairment	[68]
PD	6-OHDA mice	AT	GDNF	Intrastriatal	Enhancing TH- and NeuN-positive cell	[118]
AD	APP/PS1 mice	BM	TREM2	Intraventricular	Improvement of learning and memory function by upregulation of TREM2 and DAP12 gene expression	[212]
AD	Amyloid β rat	BM	BDNF	Intraventricular	Improving cognitive function	[69]
AD	APP/PS1 mice	BM	let-7f-5p	Intraventricular	Extending the retention time of MSCs in brain	[213]
PD	MPTP mice	AT	miR-188-3p	Intravenous	Hindrance of autophagy and pyroptosis process by downregulation of CDK5 and NLRP3	[155]
PD	Rotenone rat	UC	VEGF	Intrastriatal	Attenuation of dopaminergic neuron loss	[120]
ALS	S SOD1^G93A^ mice	UC	BDNF	Intrathecal	Improved lifespan by promotion of motor functions	[72]
PD	6-OHDA rat	WJ	PARKIN	Intrastriatal	Downregulation of the expression of c-JUN, PUMA, AIF, and caspase-3, and maintaining the mitochondrial ΔΨm, thereby inducing neuroprotective effect	[214]
PD	6-OHDA rat	BM	Neurturin	Intrastriatal	Enhancing dopamine synthesis and eliciting dopaminergic neuron protection	[215]
PD	6-OHDA rat	BM	Persephin	Intrastriatal	Promoted levels of dopamine in the striatum	[216]
PD	MPTP monkeys	BM	BDNF	Intrastriatal	Promoted levels of dopamine in the striatum	[70]
HD	YAC128 mice	BM	BDNF	Intrastriatal	Decreased striatal atrophy	[71]

MSCs, mesenchymal stromal cells; AD, Alzheimer’s disease; PD, Parkinson’s disease; ALS, amyotrophic lateral sclerosis; HD, Huntington’s disease; BM, bone marrow; UC, umbilical cord; AT, adipose tissue; WJ, Wharton’s jelly; 6-OHDA, 6-hydroxydopamine; MPTP, 1-methyl-4-phenyl-1,2,3,6-tetrahydropyridine; Aβ, amyloid beta peptide; SVZ, subventricular zone; GDNF, glial cell line-derived neurotrophic factor; BDNF, brain-derived neurotrophic factor; TH, thymidine hydroxylase; VEGF, vascular endothelial growth factor; IGF-1, insulin like growth factor-1; GFAP, G fibrillary acidic protein; miRs, microRNAs; Nurr1, nuclear receptor related 1; DAP12, DNAX-activating protein of 12 kDa; NLRP3, NOD-like receptor containing pyrin domain 3; NeuN, neuronal nuclei; CDK5, cyclin-dependent kinase 5; PUMA, P53 upregulated modulator of apoptosis; AIF, apoptosis-inducing factor; TREM2, triggering receptor expressed on myeloid cells 2; CX3CL1, chemokine (C-X3-C motif) ligand 1

Recent studies also indicated that co-administration of MSCs with resveratrol, a Sirt1 activator, could be an efficient therapeutic option for AD [101]. Resveratrol enables more efficient engraftment of MSCs in the hippocampus of the AD murine model, leading to ameliorated learning and memory, heightened neurogenesis, and reduced neural loss [101]. Furthermore, pretreatment with melatonin has been suggested as an effective strategy to prevent the low survival rate of MSCs following administration [102, 103]. Meanwhile, systemic injection of pretreated AT-MSCs with melatonin (MT-AT-MSCs) decreased Aβ levels and amended learning, memory, and cognition more evidently than AT-MSC therapy in an AD mouse model [102]. Likewise, MT-BM-MSCs showed superiority over BMSCs in terms of improvement of learning, cognition, and memory in a rat model of AD [103].

Secreted microRNAs from MSCs also widely contribute to MSC-mediated therapeutic outcomes in preclinical models of neurodegenerative diseases [104]. In 2020, Nakano and coworkers observed that intraventricular administrated BM-MSCs decreased nuclear factor kappa B (NF-κB) expression, while improving microRNA (miR)-146a levels in the hippocampus in a mouse model of AD [105]. The injected cells firstly migrated to the choroid plexus in the lateral ventricle and released miR-146a. Reduced levels of NF-κB in correlation with improved levels of miR-146a, in turn, induced astrocyte activation. Given the astrocytes’ positive roles in synapse generation, their activation ultimately induced synaptogenesis and thereby supported cognitive impairment in vivo [105].

Also, genetically modified MSCs create a paradigm shift in the neurodegenerative disease therapy. Several reports suggested that lin28B, an RNA-binding protein, could improve MSC proliferation and migration and also preserve MSCs against Aβ-induced cell death. Owing to this fact, Wu et al. transplanted lin28B-overexpressing MSCs into an AD animal model [106]. Lin28B considerably induced MSC expansion and favored their retention in vivo. Modified MSCs also reduced cognitive decline, boosted Aβ clearance, and attenuated microglia activation as well as neuronal cell apoptosis by upregulation of the insulin-like growth factor 2 (IGF2)-elicited signaling axis [106]. Likewise, anti-inflammatory cytokine chemokine (C-X3-C motif) ligand 1 (CXC3L1) and Wnt3a-overexpressing BM-MSCs ameliorated learning and memory deficits in APP/PS1 mice upon intraventricular injection [107]. Modified cells’ transplantation led to the suppression of microglial neurotoxicity and simultaneously provoked hippocampal neurogenesis by influencing the survival-involved phosphoinositide 3-kinases (PI3Ks)/AKT signaling axis [107]. Also, intraventricular injection of BM-MSCs modified to overexpress BDNF [69] or VEGF [68] could restore cognitive impairment by stimulating neurogenesis in an AD murine model in vivo.

### PD

MSCs are considered a valued therapeutic option for substituting damaged cells in PD. Recent studies showed that administration of MSCs could support functional rescue in a PD murine model following migration to the damage zone, differentiation into dopaminergic neurons, and enhancement of striatal dopamine levels [108, 109]. In an MPTP-induced mouse model of PD, systemic injection of MSCs supported blood–brain barrier (BBB) integrity, averted mannose-binding lectin (MBL) infiltration at substantia nigra compacta (SNc), inhibited microglial function, and counteracted dopaminergic neuron loss [110]. However, MSCs did not significantly differentiate into dopaminergic neurons, while secreting anti-inflammatory transforming growth factor beta 1 (TGF-β1) in SNc [110]. Also, secretion of proteinases such as matrix metalloproteinase (MMP) is another accepted mechanism applied by MSCs in PD animal models [111]. Indeed, MSCs could release MMP-2 upon systemic administration and subsequently induce neuroprotective possession by degrading aggregated α-synuclein, which usually results in inhibiting apoptotic neural cell loss in vivo [111]. In addition to the proteinopathies, MSC-secreted MMPs could elicit preferred outcomes in liver fibrosis as a consequence of their antifibrotic competencies [112].

Similar to other types of neurodegenerative disease, secretion of NTFs, such as nerve growth factor (NGF) and neurotrophin-3 (NT-3), by MSCs following intracerebral injection could potentiate neuroprotection [113]. Similarly, Park et al. observed that systemic transplantation of AT-MSCs brought about boosted BDNF and GDNF expression, protected dopaminergic neurons, and also activate the nigrostriatal pathway in the MPTP-induced mouse model of PD [63]. In another study, intranasal administration of endometrium-derived MSCs (EnSCs) ameliorated the PD symptoms in 6-OHDA induced mice [114]. Notably, the expression of nestin and thymidine hydroxylase (TH) as a differential neuronal biomarker and dopaminergic neuron marker, respectively, in SNpc delivered further proof of the hypothesis that MSCs could establish neural cell-like cells in vivo [114]. Moreover, the study of the MSC-induced beneficial effects upon intravenous or intrastriatal administration implied that intrastriatal injected cells had short-term effects on dopaminergic response in PD mouse model, whereas systemic injection had neither short-term nor long-term effects [115]. Moreover, arterially injected MSCs did not exhibit neurorestorative effects in a PD animal model in vivo [116]. These studies have outlined the importance of optimizing the administration route to achieve better outcomes in vivo [115, 116].

Apart from native MSCs, transplantation of genetically modified MSCs could also induce therapeutic outcomes in PD. Correspondingly, GDNF-MSCs suppressed neuroinflammation, inhibited neurodegeneration, and ameliorated behavioral deficits in the lipopolysaccharide (LPS)-induced PD rat model [117]. GDNF-MSCs intrastriatal administration led to the generation of condensed regions of TH-positive cells around the transplant site in vivo, reflecting the neurotrophic competence of GDNF in the LPS-induced model of PD [117]. Further, intrastriatal injection of GDNF-AT-MSCs provoked an enhancement in TH- and NeuN-positive staining in vivo and consequently restored behavioral impairment, according to a report by Sun et al. [118]. Additionally, intrastriatal administration of nuclear receptor-related 1 (Nurr1)-BM-MSCs in 6-OHDA mice [119], BDNF-BM-MSCs in MPTP-induced monkeys [70], and VEGF-UC-MSCs in rotenone-induced rats [120] elicited encouraging outcomes mainly by averting dopaminergic neurons loss accompanied with inhibiting the inflammatory response and microglial activation in vivo.

Also, combination therapy with MSCs and other molecules or modalities could heighten MSC-mediated neuroprotection in vivo [121–123]. In this regard, dextran-coated iron oxide nanoparticles showed the capability to enhance the remedial impacts of MSCs in a PD animal model [121]. This effect was robustly achieved by three main mechanisms: (1) enhancing MSC migration into the lesioned site, (2) promoting MSC differentiation into dopaminergic neurons, (3) attenuating host dopaminergic neuron loss [121]. Combination therapy with electroconvulsive therapy (ECT) and BM-MSCs also exhibited a synergistic impact against PD in MPTP mice, which was most probably caused by elevated levels of dopamine and also attenuated pro-inflammatory cytokines levels, ensuring restored functional defect [122]. ECT could increase MSC differentiation in dopaminergic neurons in vivo, and thereby may underlie more favorable therapeutic impact when used in combination with MSC therapy [122]. Moreover, the combined use of BM-MSC and granulocyte colony-stimulating factor (G-CSF), an accepted inducer of MSC proliferation, could weaken pro-inflammatory cytokines levels and also trigger antioxidant enzymes functions (e.g., SOD), and eventually raise neurogenesis in PD animal models [123].

### HD

Like other neurodegenerative conditions, MSC-based therapies have exerted therapeutic benefits and mitigated symptoms of HD in preclinical models. Importantly, secretion of stem cell factor (SCF) by damaged striatum was found to heighten engraftment of MSCs within the damaged brain [124]. In 2021, Bayat and coworkers showed that intrastriatal transplantation of olfactory ecto-mesenchymal stromal cells (OE-MSC) caused a substantial attenuation in microglial activation and TNFα expression, and also reduced necroptosis in the striatum in the neurotoxin 3-nitropropionic acid (3NP)-induced rat model of HD [125]. Similarly, in quinolinic acid (QA)-induced rat model of HD, BM-MSC administration ameliorated motor dysfunction and simultaneously sustained striatal volume [126]. The encouraging outcomes might be attributable to MSC-mediated paracrine effects, which were mainly inspired by upregulated levels of NGF, BDNF, GDNF, and ciliary neurotrophic factor (CNTF) in the striatum of treated animals [126]. These findings have conferred the critical role of NTFs in MSC-induced neuroprotection in vivo. In another study, in the R6/2 mouse model of HD, transplanted BM-MSCs cells survived, and treated animals experienced remarkable behavioral and morphological sparing compared with control animals as a result of improved BDNF levels in striatum post-transplantation [127]. These observations implied that the duration of time that the MSCs are exposed to in vitro culture circumstances might modify their efficacy in vivo. Further, GDNF and VEGF secreted by UC-MSCs were found to contribute to compromising the disapproving influence of oxidative stress in the HD rat model [66]. Accordingly, in a 3-NP-induced rat model of HD, bilateral striatal administration of UC-MSCs improved neural cell viability and neurite outgrowth, diminished gliosis, and restored motor coordination and muscle activity in vivo [66]. Also, intervention gave rise to significant enhancement in striatal volume as well as the dendritic length of the striatum in treated animals [66]. However, Rossignol et al. suggested that GDNF and CNTF were not significantly involved in MSC-mediated favored impact in 3NP rats [128]. They supposed that secretion of BDNF in association with enhanced collagen type I and fibronectin in the brains of MSC-transplanted rats mainly enhances their behavioral sparing. Also, the conducted study did not verify MSC differentiation into the neural cell in vivo [128].

Although the intrastriatal transplantation of stem cells has been shown to have a valuable influence in murine models of HD, the aggressive nature of the surgical process accompanied by its capacity to provoke the host immune response can hinder its clinical application. Therefore, scientists have focused on the development of a low-invasive administration route. Meanwhile, intranasal administration of BM-MSCs led to improved survival rate and restored circadian activity, as evidenced by the assessment of locomotor activity in R6/2 HD transgenic mice [129]. Moreover, MSCs were recognized in the olfactory bulb, midbrain, and striatum, and boosted the dopamine- and cAMP-regulated phosphoprotein, Mr 32 kDa (DARPP-32), and TH protein expression in vivo, leading to ameliorated phenotypes of treated mice [111]. Thereby, intranasal administration could be an alternative route for MSC therapy in HD [129]. Also, some studies were carried out to evaluate the beneficial effects of preconditioned MSCs or combination therapy with MSCs and other molecules or modalities in HD rat models [130, 131]. Correspondingly, Elbaz et al. found that intraperitoneal injection of lipophilic calcium antagonist, lercanidipine (LER), in combination with systemic injection of BM-MSCs could support better functional recovery in treated rats [130]. This regimen also downregulated inflammation, reduced Bax/Bcl2 ratio, and conversely raised BDNF, forkhead box P3 (FOXP3), Wnt, and β-catenin protein expression in the striatum of treated models, restoring striatum tissue damages [130]. Moreover, preconditioned MSCs with mood stabilizers lithium and valproic acid (VPA) potentiated the therapeutic impacts of such stromal cell therapy in N171-82Q HD transgenic mice more evidently than administration of non-preconditioned MSCs [131]. Intranasal injection of preconditioned MSC caused better motor function as well as attenuated striatal neuronal loss and HTT assemblies than transplantation of non-preconditioned MSCs in HD mice [131]. Moreover, preconditioned stromal cells experienced better survival than non-preconditioned cells in vivo [131]. Finally, there is clear evidence confirming that intrastriatal transplantation of modified BM-MSCs to overexpress BDNF could significantly decrease anxiety and striatal atrophy, augment neurogenesis, and ultimately improve the overall survival of mice model of HD in vivo [71].

### ALS

Studies have shown that MSCs could have a beneficial influence on ALS symptoms thanks to their great competencies to secrete a diversity of NTFs, ranging from BDNF and VEGF to GDNF [132]. In vivo, intraspinal and systemic administration of MSCs ameliorated the ALS course and partially prolonged the overall survival of treated rodents (about 190 days in the treated group versus 179 days in the control group) [133]. Treated rodents experienced improved motor function along with a greater population of motor neurons at the thoracic and lumbar levels [133]. In another study, tracking of human skeletal muscle-derived stem cells (SkmSCs) was accomplished post-transplantation with superparamagnetic iron oxide (SPIO) nanoparticles and Hoechst 33258 in the ALS mouse model [134]. Accordingly, Canzi et al. showed that interventions led to improved motor function and reduced inflammatory cytokine expression concomitant with significant protection of functional neuromuscular junctions in vivo [134]. However, the intervention did not cause a decrease in motor neuron death at the cervical spinal cord, and only modest injected stem cell integration in the brain parenchyma was observed. However, these observations reinforce the premise of the conceivable association between inflammation, cytotoxicity, and ALS [134]. Also, systemic administration of AT-MSCs exerted neuroprotection by induction of a shift in the secretome of local glial cells toward a neuroprotective phenotype in the SOD mouse model of ALS. The observed neuroprotection was mainly mediated by FGF but not GDNF, underlining the durable influence on motor function in treated mice [135]. Another important study in SOD1^G93A^ ALS mice demonstrated that MSC systemic infusion could lead to prolonged survival and motor activity in vivo [136]. Also, MSC therapy elicited antioxidant impacts, diminished ubiquitin agglomerates, and downregulated both astrocyte and microglia activation in the spinal cord of treated animals, supporting the justification for their use to treat ALS [136]. Besides, comparing the intraventricular administration of MSCs versus single and repeated intraspinal administration of such cells was managed in ALS mice by Bursch and coworkers [137]. They showed that intraspinal administration slightly improved overall survival in treated mice, while MSC delivery by intraventricular route surprisingly stimulated microgliosis and robustly attenuated overall survival of the treated animal. Moreover, injected MSCs were observed at the administration area on day 20 after intraspinal injection but no longer on day 70, signifying that MSC transplantation by intraspinal route can be a reasonable plan for ALS therapy compared with the intraventricular administration [137]. Besides, intrathecal concomitant with intramuscular injection of MSC in SOD1 ^G93A^ rats meaningfully augmented survival, ameliorated motor dysfunction, and downregulated necroptosis apoptosis and autophagy process [138]. Importantly, reduction in astrogliosis, as well as Connexin 43 levels post-transplantation provided a new indication for the combination of repeated intrathecal and intramuscular administration of MSC exerting motor neuroprotection and supporting neuromuscular junctions in ALS animal models [138]. In this regard, other reports also showed that intrathecal injection of MSCs has no effect on astrogliosis in the ALS rat model, while such cells gave rise to astrocytes at degeneration regions post-transplantation [139]. The MSC-derived healthy astrocytes in vivo reduced motor neuron loss in the lumbar spinal cord, thereby potentiating motor functions and improving the lifespan of treated mice [139]. The favored outcomes were probably stimulated by reduced microglial activation and downregulation of the expression of cyclooxygenase-2 (COX-2) and NADPH oxidases-2 (NOX-2), supporting antioxidant and anti-inflammatory microenvironment [139]. Other studies also suggested that the shift from a pro-inflammatory (IL-6, IL-1β) to an anti-inflammatory (IL-4, IL-10) and neuroprotective (IGF-1) environment in the lumbar spinal cord following UC-MSC therapy may be due to the stimulation of Akt survival signaling axis in motor neurons as well as in reactive astrocytes in SOD1^G93A^ mice [59].

Recently, Van Dyke and colleagues pointed out that human GDNF-overexpressing MSCs improved survival and supported neuromuscular junction and also motor neurons in SOD1^G93A^ following injection into limb muscles [140]. In vivo, intramuscular injection of GDNF-overexpressing MSC reduced inflammation and considerably sustained neuromuscular junction [140]. Also, transplantation of genetically modified UC-MSC-derived motor neurons to overexpress BDNF improved survival and restored motor function of the treated ALS mice [72]. It was found that motor-neuron-related marker expression, such as acetyltransferase (ChAT) and homeobox protein 9 (HB9), was improved in the ALS group. Also, intervention-associated effects, in addition to the motor neuron activities, depended on the upregulated expression of BDNF in vivo [72].

## Preclinical studies based on MSC-derived exosome therapy in neurodegenerative diseases

Exosomes, as an emergent approach to mediate intercellular communication, deliver innovative viewpoints on known therapeutic tactics for neurodegenerative diseases. MSC-derived exosomes, owing to their minimal immunogenicity and tumorigenicity as well as easy storage, have devoted accumulating attention and led to promising results in vivo (Table 3).

**Table 3:** MSC-derived secretome (e.g., exosome) therapy in preclinical models of common neurodegenerative diseases

Condition	Model	Cell source	Administration route	Results	Ref.
AD	3xTg mice	BM	Intranasal	Induction of neuroprotection by provoking anti-inflammatory effect	[92]
AD	APP/PS1 mice	BM	Intravenous	Amelioration in learning and memory capabilities, reducing Aβ levels and supporting anti-inflammatory environment	[217]
AD	APP/PS1 mice	BM	Intravenous	Cognitive impairment rescue by improving microRNA-146a levels in the hippocampus	[105]
AD	Aluminum chloride-injected rat	BM	Intravenous	Pathological symptoms rescue	[218]
PD	6-OHDA rat	BM	Intravenous	Attenuation of dopaminergic neuron loss in SN, and improving dopamine levels in the striatum	[93]
PD	6-OHDA rat	BM	Intravenous	Neuroprotective effect on dopaminergic neuron	[219]
PD	MPTP mice	AT	Intravenous	Suppression of autophagy and pyroptosis	[155]
PD	MPTP mice	AT	Intraperitoneal	Promotion of the angiogenesis of human brain microvascular endothelial cells (HBMECs)	[95]
PD	6-OHDA rat	AT	NA	Induction of neuroprotection by upregulation of the sirtuin 3 (SIRT3) levels	[220]
PD	6-OHDA rat	BM	Intravenous	Improving motor function	[221]
PD	6-OHDA rat	BM	Intranigral Intrastriatal	Reverting motor phenotype and the neuronal organization	[222]
PD	α-Syn induced *C. elegans*	BM	NA	Reduced number of α-syn inclusions	[153]
HD	R6/2 mice	AM	Intraperitoneal	Amelioration of neurological complications rotarod function	[162]
ALS	SOD1^G93A^ mice	AT	Intravenous Intranasal	Inhibition of glial cells activation, supporting motor neuron rescue, and protecting the neuromuscular junction	[161]

MSCs, mesenchymal stromal cells; AD, Alzheimer’s disease; PD, Parkinson’s disease; ALS, amyotrophic lateral sclerosis; HD, Huntington’s disease; BM, bone marrow; 6-OHDA, 6-hydroxydopamine; MPTP, 1-methyl-4-phenyl-1,2,3,6-tetrahydropyridine; Aβ, amyloid beta peptide; AM, amniotic membrane

### AD

Several studies have shown that MSC-derived exosomes could attenuate Aβ expression and conversely upregulate neuronal memory/synaptic plasticity-related gene expression in AD in vitro and in vivo models [141]. These alterations, in turn, augment brain glucose metabolism and also alleviate cognitive dysfunctions in AD transgenic mice [141]. As well, MSC-derived exosome could upregulate anti-inflammatory mediators such as IL-10 or tissue inhibitor matrix metalloproteinase 1 (TIMP1) in activated microglia, eliciting anti-inflammatory responses in AD animal models [142]. Meanwhile, it seems that various molecules in MSC-derived exosome play a more important role in exosome-mediated neuroprotection. Meanwhile, increasing research has focused on the roles of miRNAs [24, 105, 143].

Recent studies have shown that miR-223-enriched MSC-derived exosome adjusts neuronal cell apoptosis in the AD in vitro model [24]. The MSC-derived exosome could diminish hypoxia-inducible factor (HIF)-1 expression, attenuate neural cell apoptosis, and boost their migration mainly by miR-223 delivery. It seems that miR-223 suppressed neuron loss in vitro through downregulation of phosphatase and tensin homolog (PTEN), leading to upregulation of survival-involved PI3K/Akt pathway [24]. Also, exosomal miR-146a released from BM-MSCs could downregulate inflammation-involved NF-κB pathways in astrocytes and then restore astrocytic activation, leading ultimately to improved synaptogenesis and ameliorated cognitive deficits in AD model mice [105]. Also, miR-29 enriched MSC-derived exosome therapy resulted in decreased pathological impacts of Aβ peptide in a rodent model of AD and then improved spatial learning and memory [143]. On the other hand, miRNA-22-loaded AT-MSC-derived exosomes enhanced the motor and memory capability of mice model of AD by improving neural survival in vivo [144]. In vitro analysis also suggested that improved neural survival relies on the inhibition of inflammatory factors secretion and downregulation of the pyroptosis process, which was provoked by exosomal miRNA-22 [144].

Another study revealed that thrombospondin-1 (TSP1)-containing UCB-MSC-derived exosome restored synaptic dysfunction in AD rodent model in vivo [145]. In fact, TSP-1-enriched exosome could ameliorate synaptic impairment in vitro and in vivo, as shown by enhancement in synaptic-density marker levels, including synaptophysin (SYP) and post-synaptic density protein-95 (PSD-95) [145]. Similarly, Wang et al. verified the existence of a correlation between promoted cognitive behaviors and improved synaptic transmission with suppressed iNOS expression after MSC-derived exosome therapy in AD mouse models [146]. Indeed, they found that downregulation of iNOS expression is another mechanism applied by such exosomes to rescue neural impairment in vivo [146]. Besides, reduced expression of p53, Bax, pro-caspase-3, and cleaved-caspase-3, and conversely improved expression of Bcl-2 was revealed following AT-MSC-derived exosome therapy in a transgenic mouse model of AD [147]. Like other reports, this study reflects the promising potential of MSC-derived exosome to inspire pro-survival effects against Aβ-triggered neuronal death in AD [147]. Further, Katsuda and colleagues proved the existence of neprilysin, the most pivotal Aβ-degrading enzyme, in AT-MSC-derived exosome [148]. In vitro, neprilysin-containing exosome reduced both released and intracellular Aβ levels in N2a cells, a fast-growing mouse neuroblastoma cell line, more efficiently than BM-MSC-derived exosome, representing the beneficial significance of AT-MSCs exosomes for AD [149]. In another study, BM-MSC-derived exosomes suppressed the levels of Aβ1-40, Aβ1-42, β-amyloid precursor protein cleaving enzyme (BACE1), and presenilin 1 (PS1), while upregulating NeuN expression in cortex and hippocampus of treated mice, which led to improved cognitive impairment [150]. Notably, upregulation of survival and proliferation-inducing sphingosine kinases (SphKs)/sphingosine-1-phosphate (S1P) signaling pathway was found to play a crucial role in this regard [150]. Besides, MSC-derived exosome, owing to the presence of high levels of GDF-15, is a rational candidate for AD therapy. Accordingly, Kim et al. found that UCB-MSCs, because of the existence of GDF-15, enhanced Aβ plaque clearance by upregulation of both insulin-degrading enzyme (IDE) and neprilysin expression in microglial cells in vivo, clarifying another therapeutic appliance for AD [151]. Indeed, GDF-15 improves neprilysin and IDE expression as well as activation by stimulation of AKT/GSK-3β/β-catenin pathway, thus degrading Aβ peptide [152].

### PD

Recent studies have shown that pretreatment with MSC-derived exosome could improve 6-OHDA-stimulated SH-SY5Y cells to expand and then dampen apoptosis through stimulating autophagy and also stimulate neuroprotective effects [93, 94]. In vivo, transplanted exosomes could reach the SN by the blood–brain barrier (BBB), diminished apoptosis of dopaminergic neurons in SN, and simultaneously enhanced dopamine levels in treated rodents’ striatum [93]. In a PD mouse model, Xue et al. also found that MSC-derived exosome stimulated the angiogenesis of human brain microvascular endothelial cells (HBMECs) following enhancing the intercellular adhesion molecule-1 (ICAM-1) expression and also restoring 1-methyl-4-phenylpyridinium (MPP^+^)-induced damage on their endothelial cells [95]. Indeed, upregulation of ICAM1 by MSC-derived exosome could trigger HBMEC angiogenesis by provoking the SMAD3 as well as P38MAPK signaling pathways in PD animal models [95]. Moreover, intraperitoneal injection of exosomes could strikingly improve TH-expressing positive cells in SN of treated rodents, and upregulated CD31 expression in the corpus striatum area in vivo, stimulating PD recovery [95]. Besides, BM-MSC secretome (containing exosome) inspired a neuroprotective influence and consequently lessened dopaminergic neurodegeneration in a *Caenorhabditis elegans* model of PD [153]. Moreover, intervention decreased α-syn aggregates, suggesting that injected MSC-derived secretome can underlie degradation of such structures. Further, in silico investigations recognized conceivable inhibitors of α-syn proteotoxicity, most importantly, growth factors [153]. In another study in the 6-OHDA-induced rat model of PD, MSC-derived secretome alleviated neurobehavioral deficits and reduced inflammation, oxidative stress, and apoptosis [154]. Further studies to elucidate the underlying mechanism behind the MSC-derived exosome-induced anti-inflammatory and anti-apoptotic effects in the PD rodent model highlighted the importance of miR-188-3p [155]. The miR-188-3p-containing MSC-derived exosome could inhibit autophagy and pyroptosis while stimulating proliferation by inhibition of cyclin-dependent kinase 5 (CDK5)-induced autophagy and NLRP3-induced inflammation in treated rodents and also MN9D cells [155]. Moreover, it has been suggested that the antioxidant effect, which is elicited by MSC-derived secretome, could bring about by Sirt3 delivery, leading to substantial neuroprotective influences in vivo [156]. Other reports have also shown that desired effects induced by MSC-derived exosome therapy were largely mediated by various biologic molecules in MSC-derived secretome, including stromal cell-derived factor-1 (SDF-1 or CXCL12), growth factors (BDNF, VEGF and GDNF), MMP2, heat shock protein 27 (HSP27), and semaphorin 7a (sema7a) [157].

### ALS and HD

Studies have shown that AT-MSC-derived exosome could play a considerable neuroprotective role in ALS in vitro models [158]. The analysis discovered about 189 proteins in AT-MSC-derived exosome, largely contributing to the cell adhesion and negative modification of the apoptotic pathways. It seems that exosome therapy could suppress the expression of pro-apoptotic proteins Bax and cleaved caspase-3 and conversely improve the expression of anti-apoptotic protein Bcl-2 in ALS in vitro models [158]. Thereby, MSC-derived exosomes could be applied as an innovative tactic for neurodegenerative disease therapy. Moreover, a recent in vitro study revealed that AT-MSC-derived exosome could protect NSC-34 cells overexpressing human SOD1^G93A^ from oxidative stress [159]. It was suggested that the beneficial impact of AT-MSC-derived exosome therapy was mediated by various miRNAs, including miRNA21, miRNA222, and miRNAlet7. Such miRNAs negatively regulate the apoptosis axis, promote cell growth as well as proliferation, and exert neuroprotective effects [160]. Also, intravenous and intranasal administration of AT-MSC-derived exosome in the SOD1^G93A^ mouse model of ALS was performed by Bonafede and coworkers [161]. They found that repeated injection of such exosomes suppressed glial cell functions, restored motor dysfunctions, and supported lumbar motoneurons, NMJs, and muscle up to 17 weeks post-transplantation in vivo [161]. In 2019, Giampà et al. also illustrated that conditioned medium isolated from amniotic membrane-derived MSCs prompted neuroprotective effects in vitro and in the R6/2 mouse model of HD, with diminished impairments in rotarod function [162]. The injected conditioned medium also decreased striatal atrophy, suppressed microglial activation, and downregulated iNOS levels, while surprisingly having no impact on BDNF levels post-transplantation in vivo [162].

In sum, the exosomes orchestrate various events that, in turn, facilitate recovery and regeneration in neurodegenerative conditions. Much effort has been spent on improving the homing property of MSC-derived exosomes to convey molecular agents to brain lesions and potentiate recovery. Merging the intrinsic attributes of the exosomes with a targeted medication is speculated as a novel therapeutic strategy that might have a substantial influence on the future of neurodegenerative disease treatment.

## Clinical trials

### AD

Although various clinical trials have been conducted or are ongoing to address the safety and efficacy of MSC therapy in AD (e.g., NCT02833792, NCT04040348, NCT01547689, NCT04482413, and NCT04388982), few reports have been published. A study of the possible effects of the administration of allogeneic UCB-MSCs to the hippocampus and precuneus by stereotactic injection in patients with AD was carried out by Kim et al. (NCT01297218 and NCT01696591) [163]. In this trial, patients received 60 µL cell suspension containing 3.0 or 6.0 × 10^6^ cells. The intervention had no unwanted stern events and also dose-limiting toxicity during 2 years of follow-up. The common untoward events were wound pain from the surgical procedure, headache, dizziness, and postoperative delirium. Thereby, the safety and feasibility of MSC transplantation were confirmed [163]. Another phase I clinical trial in nine patients with mild-to-moderate AD also indicated that intracerebroventricular (ICV) transplantation of human UCB-MSCs (1.0 or 3.0 × 10^7^ cells/2 mL) could be safe and feasible [164]. This trial was conducted in Samsung Medical Center, Seoul, Republic of Korea, and all patients received three repeated transplantations of MSCs at 4-week intervals. The common untoward events were fever, headache, nausea, and vomiting, which were alleviated within 36 h [164]. Moreover, an important clinical trial on ten patients with AD, six patients with ALS, six patients with progressive multiple sclerosis, six patients with PD, one patient with spinal cord injury, one patient with traumatic brain injury (TBI), and one patient with a stroke was accomplished to assess the safety of human ICV brain administration of autologous adipose-derived stromal vascular fraction (ADSVF) [165]. The transient meningioma and mild fever were the most common adverse events, circumvented with acetaminophen and/or dexamethasone. This study delivers further data for documentation of the safety of MSC-based therapies in patients with neurodegenerative diseases, such as AD [165].

### PD

Several trials have been conducted or are ongoing, aiming to determine the safety and efficacy of MSCs administration in PD (e.g., NCT02611167, NCT03684122, NCT04146519, NCT00976430, NCT04506073, and NCT04388982). In 2009, for the first time, a trial performed by Venkataramana et al. verified the safety and efficacy of unilateral administration of autologous BM-MSCs in seven patients with PD. During 10–36 months follow-up, three of seven participants exhibited steady enhancement in their “off”/“on” Unified Parkinson’s Disease Rating Scale (UPDRS) [166]. Also, Hoehn and Yahr (H&Y) and Schwab and England (S&E) scores presented significant enhancement from 2.7 and 2.5 in H&Y and 14% enhancement in S&E scores, respectively. A remarkable amelioration was also shown in symptoms, such as facial expression, gait, and freezing episodes [166]. In 2020, Carstens and colleagues also found that intranasal and intramuscular (into facial muscle) administration of autologous adipose-derived stromal vascular fraction (SVF) could lead to promising outcomes in patients with PD [167]. Meanwhile, two participants with PD received 6 × 10^6^ total nucleated cells in processed SVF. Observations evidenced amelioration in motor and nonmotor symptoms post-transplantation. Also, on-medication UPDRS motor scores were reduced in both enrolled patients. Owing to its unfamiliar mechanism of action, this treatment authorizes cautious verification and examination [167].

### ALS

Various reports have documented the safety of MSC therapy in patients with ALS [168, 169]. In 2003, Mazzini et al., for the first time, evaluated the feasibility and safety of intraspinal injection of autologous BM-MSCs in patients with ALS [168]. With the exception of modest intercostal pain irradiation, and leg sensory dysesthesia, no patients experienced other serious unwanted events [168]. Moreover, no symptoms of abnormal cell growth were found in the spinal cord, suggesting that the intervention was safe and well tolerated [168]. Similarly, another phase I clinical trial conducted by Mazzini and coworkers in 19 patients with ALS showed that transplantation of autologous BM-MSC was safe and feasible while having no significant encouraging therapeutic outcomes [169]. No structural changes such as tumor formation or worsening in the psychosocial status were presented post-transplantation [169]. On the other hand, there is evidence indicating that repeated transplantation of autologous MSCs could avert disease progress in patients with ALS with an inherently rapid course [170]. In addition to the intraspinal administration, the safety and feasibility of intrathecal transplantation of BM-MSCs were proven in patients in ALS, with no significant efficacy [171]. In another trial carried out between June 2011 and October 2014, intrathecal and intramuscular transplantation of MSC-secreting NTF cells was safe and also caused modest clinical benefits in patients with ALS [172]. Moreover, Kim et al. found that VEGF, angiogenin, and TGF-β levels in MSCs could be exploited as capable biological markers to predict the efficacy of intrathecal injection of MSCs in patients with ALS [173].

There are three ongoing trials to address the safety and efficacy of MSC therapy in patients with ALS (e.g., NCT04651855, NCT05003921, NCT02290886, and NCT03296501); however, there are no reliable registered trials on MSC therapy in patients with HD.

## Conclusion and future direction

**Fig. 3:**
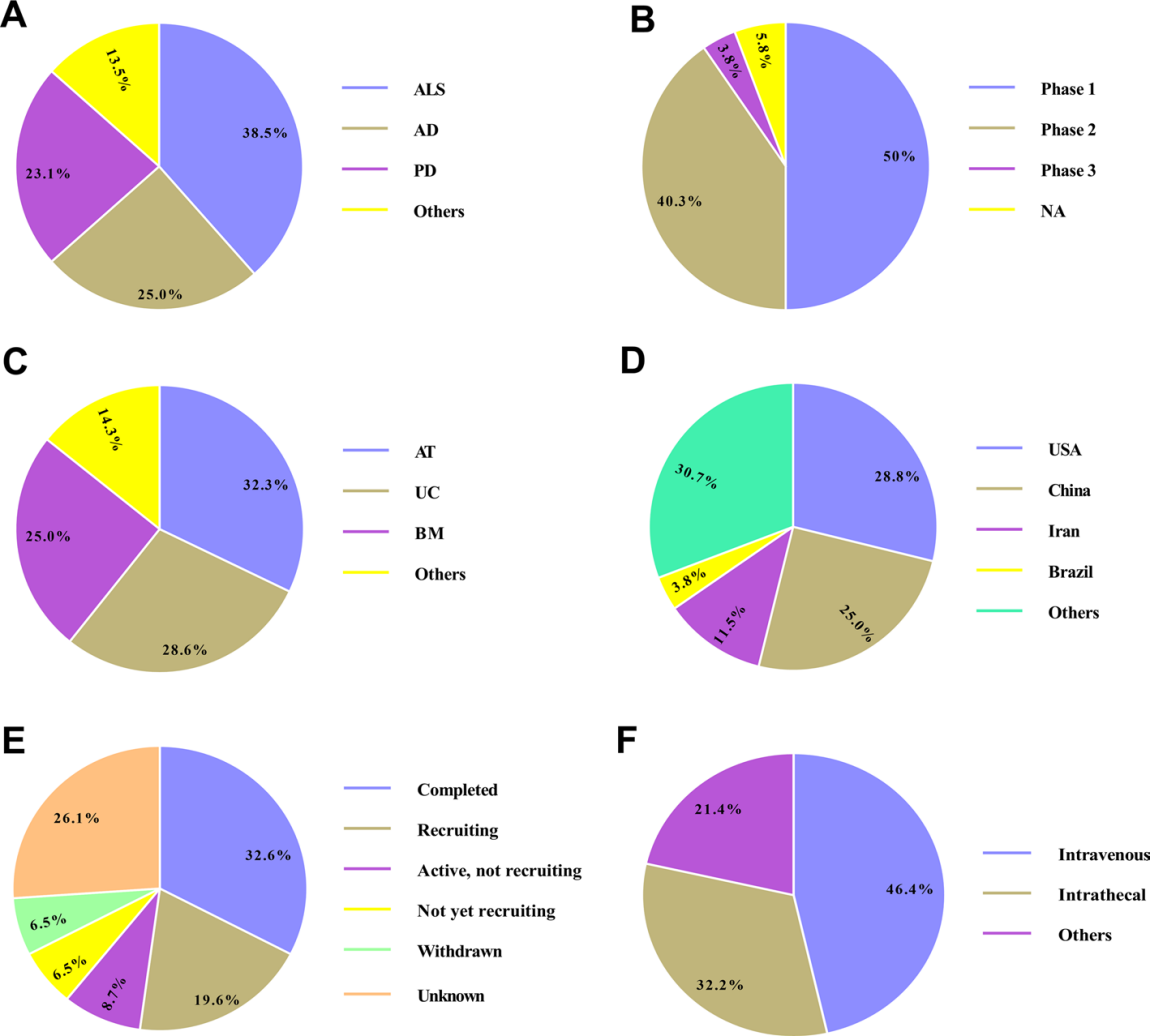
Clinical trials based on mesenchymal stromal cell (MSC) therapy in common neurodegenerative diseases registered on ClinicalTrials.gov (April 2022). The schematic demonstrates clinical trial according to condition (**A**), phase (**B**), MSC source (**C**), study location (**D**), study status (**E**), and administration rote (**F**)

A myriad of clinical trials have been conducted to address the safety and efficacy of MSC transplantation in patients with neurodegenerative diseases (Table 4) (Fig. 3). The intravenous and intrathecal routes are the most common transplantation routes in these patients. Despite promising therapeutic outcomes in animal models, MSC therapy has conferred no remarkable effectiveness in patients suffering from neurodegenerative diseases. Of course, the safety and feasibility of transplantation of both autologous and allogeneic MSCs (BM, AT, UC, or others) have been documented [166, 168, 169, 174]. On the basis of published reports, no tumors were formed because of the transplant, and no deaths occurred because of the therapeutic intervention. As various neurodegenerative disorders result from abnormalities in several molecular pathways and dissimilar cell types, it is predictable that the trophic range of molecules makes it difficult to sustain the neuronal and glial activity. Although multiple favorable factors influencing the important competencies of neurodegenerative disorders have been well defined, the potential of MSC-based treatments is only beginning to unravel, as clinical trials administrating these cells meet safety criteria. Like other innovative therapies, the administration of MSCs will face some unexpected outcomes. For example, systematic translational use of MSC therapy is still somewhat out of reach [175]. Also, it is of paramount importance to note that there is variability in cell yield, survival, and the differentiation competencies among MSCs isolated from dissimilar sources [176]. MSCs produced from UC proliferate more rapidly than MSCs derived from AT, while AT-derived MSCs proliferate more quickly than BM-MSCs. Also, AT-MSCs have superiority over stem cells derived from other sources in terms of generating synaptic structures, making them an ideal source for PD therapy [177].

**Table 4:** Summary of important clinical trials based on MSC therapy in common neurodegenerative diseases (April 2022)

Condition	Phase	Cell source	Administration route	Participant number	Status	Location	NCT number
AD	1	UCB	NA	9	Completed	South Korea	NCT01297218
AD	2	UC	Intravenous	40	Recruiting	USA	NCT02833792
AD	1	UC	Intravenous	6	Recruiting	USA	NCT04040348
AD	2	AT	Intravenous	80	Not yet recruiting	South Korea	NCT04482413
AD	1/2	AT-exosome	Intranasal	9	Recruiting	China	NCT04388982
AD	1/2	AT	Intravenous	21	Completed	USA	NCT03117738
AD	1/2	UC	Intravenous	24	Recruiting	South Korea	NCT02899091
AD	1/2	UCB	Intraventricular	46	Completed	South Korea	NCT02054208
PD	1	BM	Intravenous	20	Completed	USA	NCT02611167
PD	1/2	UC	Intrathecal Intravenous	10	Active, not recruiting	Jordan	NCT03684122
PD	NA	BM	Intrastriatal	5	Terminated	India	NCT00976430
PD	2	UC	Intravenous	45	Active, not recruiting	USA	NCT04506073
PD	1	UC	Intravenous	20	Enrolling by invitation	China	NCT03550183
PD	2/3	BM	Intravenous	50	Recruiting	Belarus	NCT04146519
PD	1	AT	Intracranial	9	Not yet recruiting	Taiwan	NCT05094011
PD	NA	AT UC	Intrathecal Intravenous	15	Recruiting	Indonesia	NCT04876326
ALS	1/2	BM	Intrathecal	20	Completed	Israel	NCT04821479
ALS	1	AT	Intravenous	19	Completed	Iran	NCT02492516
ALS	1	BM	Intrathecal	8	Completed	Iran	NCT01771640
ALS	1/2	WJ	Intrathecal	20	Recruiting	Poland	NCT04651855
ALS	1	AT	Intrathecal	27	Completed	USA	NCT01609283
ALS	1	NA	Intrathecal	3	Completed	Brazil	NCT02987413
ALS	2	BM	Intramuscular Intrathecal	48	Completed	USA	NCT02017912
ALS	3	BM	Intrathecal	263	Completed	USA	NCT03280056
ALS	1/2	AT	Intravenous	52	Active, not recruiting	Spain	NCT02290886
ALS	1	AT	Intraspinal	30	Active, not recruiting	Poland	NCT03296501

MSCs, mesenchymal stromal cells; AD, Alzheimer’s disease; PD, Parkinson’s disease; ALS, amyotrophic lateral sclerosis; BM, bone marrow; UC, umbilical cord; UCB, umbilical cord blood; AT, adipose tissue; WJ, Wharton’s jelly

Suspended trials or studies with “unknown” status are not listed

Researchers have sought different strategies to augment the efficiency of MSC-based therapy. Among them, preconditioning of MSCs (priming or genetic modification) has attracted increasing attention [75, 178–180]. Cell priming entails the exposure of cells to growth conditions to mimic the in vivo microenvironment of injured tissue [181]. MSCs, in fact, modify their cellular signaling in reaction to primed culture conditions, and thereby administration of the primed MSCs potentiates their function, survival, and therapeutic efficacy. Multiple priming strategies have been investigated, in particular priming with inflammatory cytokines or mediators, growth factors, and hypoxia [182]. The drawback of this plan is the restricted consensus in cell manufacturing protocols, bringing about the difficulty in attaining quality assurance for clinical-grade MSCs.

Several reports have ascertained the superiority of MSC-derived exosome on parental cells, as discussed in the previous section. Notwithstanding, exosomes are mainly a part of heterogeneous populations, and their metabolomic and lipidomic profiles have not yet been well detected [183]. Other boundaries of exosome separation and purification include the procedure itself, which involves inconsistency in the quality of exosome preparations, exosome yield, and the potential for non-exosome contaminants in the preparation [184]. Prior to utilizing exosome in clinical trials, comprehensive evaluation to determine their safety and efficacy is urgently required.

The phenotypes, produced factors, and proliferative, migratory, differentiating, and immunomodulatory potential of MSCs rely on the certain mediators that exist in their microenvironment [185–187]. Elucidating microenvironmental factors and their internal mechanisms in MSC responses may aid in the enhancement of clinical merits. Thus, the role and mechanism of several microenvironmental factors, such as IL-1α/β, TNFα, and stromal cell-derived factor 1 (SDF1 or CXCL12), which affect the MSC properties, are the emphasis of MSC clinical utility as such factors influence the treatment outcomes [188]. Through the increasingly accurate in vitro models that mimic the local tissue circumstances, the performance of MSCs or their differentiated progeny can be experimented before in vivo administration [185]. Through such strategies, influences from soluble mediators or other cell types, which can hinder the preferred therapeutic outcome, can be evaluated. Further research on microenvironmental factors must be managed to boost the therapeutic effect of MSCs. In various cases, co-therapy with a pharmacologic such as a cytokine receptor antagonist may bypass the undesired impacts of the microenvironment and optimize the therapeutic capacity of MSCs [188].

In conclusion, to offer more effective treatment approaches for patients, it is essential to improve the procedure of MSC preparation, the doses that should be used, and the administration route. Undoubtedly, recognizing such processes in more detail will promote the therapeutic evolution of MSCs and eventually potentiate their impending therapeutic effectiveness.

## Data Availability

Not applicable.

## Abbreviations

MSCs: Mesenchymal stromal cells
AD: Alzheimer’s disease
PD: Parkinson’s disease
ALS: Amyotrophic lateral sclerosis
HD: Huntington’s disease
BM: Bone marrow
UC: Umbilical cord
UCB: Umbilical cord blood
AT: Adipose tissue
GDNF: Glial cell line-derived neurotrophic factor
BDNF: Brain-derived neurotrophic factor
miRs: MicroRNAs
